# The roles of lncRNA functions and regulatory mechanisms in the diagnosis and treatment of hepatocellular carcinoma

**DOI:** 10.3389/fcell.2022.1051306

**Published:** 2022-11-18

**Authors:** Yuling Hong, Yunxing Zhang, Haibo Zhao, Hailing Chen, Qing-Qing Yu, Hongxia Cui

**Affiliations:** ^1^ School of Clinical Medicine, Jining Medical University, Jining, China; ^2^ Jining First People’s Hospital, Jining Medical College, Jining, China

**Keywords:** hepatocellular carcinoma, long noncoding RNA, gene expression regulation, biomarker, diagnosis and therapy

## Abstract

Hepatocellular carcinoma (HCC) is the most frequent and deadly type of liver cancer. While the underlying molecular mechanisms are poorly understood, it is documented that lncRNAs may play key roles. Many HCC-associated lncRNAs have been linked to HBV and HCV infection, mediating gene expression, cell growth, development, and death. Studying the regulatory mechanisms and biological functions of HCC-related lncRNAs will assist our understanding of HCC pathogenesis as well as its diagnosis and management. Here, we address the potential of dysregulated lncRNAs in HCC as diagnostic and therapeutic biomarkers, and we evaluate the oncogenic or tumor-suppressive properties of these lncRNAs.

## Introduction

Primary liver cancer is more common in males than in women and is associated with a high fatality rate worldwide (Sung et al., 2021). Viral infection (HBV, HCV), aflatoxin contamination of food, heavy alcohol use, being overweight or obese, smoking, and having type 2 diabetes have all been associated with the development of HCC (Marrero et al., 2005; Singal and El-Serag, 2015; McGlynn et al., 2021). Currently, effective treatments for HCC include targeted therapy, surgical resection, chemotherapy, radiotherapy, interventional therapy, and immunotherapy (Heimbach et al., 2018). However, since HCC is often diagnosed at a late stage, overall survival (OS) remains inadequate (Singal et al., 2022). Thus, it is crucial to improve diagnostic and therapeutic strategies to prolong survival in HCC patients and reduce the global cancer burden.

LncRNAs are RNA molecules that are 200 nucleotides or longer but do not code for proteins. Most are nuclear, with some located in the cytoplasm. The involvement of lncRNAs in tumorigenesis, specifically, as they modulate both gene expression and signal transduction, is well documented (Carlevaro-Fita and Johnson, 2019; Maass et al., 2019; Morf et al., 2020). Evidence suggests that LncRNAs have a role in every stage of HCC progression, from initial cell proliferation and differentiation through invasion, infiltration, and metastasis (Huo et al., 2017; Lim et al., 2019; Huang Z. et al., 2020). Understanding the pathophysiology of HCC and discovering important tumor markers to increase the sensitivity and specificity of HCC diagnosis and produce HCC-specific medicines may be achieved *via* research into the biological regulatory mechanisms and activities of lncRNAs. Here, we review the functions and regulatory activities of HCC-related lncRNAs and outline their involvement in the promotion or inhibition of HCC tumorigenesis and, based on this, analyze their potential for diagnosing and treating HCC (Table 1).

**TABLE 1:** Some dysregulated lncRNAs in HCC and their roles.

LncRNAs	Roles in HCC	Molecular mechanism	Functions	References
LEU2	Oncogene	LncRNA DLEU2/HBx/EZH2/PRC2	The transcription and replication of cccDNA in HBV-related HCC	Salerno et al. (2020)
PCNAP1	Oncogene	LncRNA PCNAP1/miR-154/PCNA	HBV replication and HBV cccDNA accumulation	Feng et al. (2019)
LINC01149	Oncogene	LINC01149/miR128-3p/MICA	Promoting HBV remission but increases the risk of HCC	Zhong et al. (2020)
IFI6	Oncogene	LncRNA-IFI6/IFI6	Increasing HCV replication	Liu et al. (2019)
Linc-Pint	Tumor suppressor	Linc-Pint/SRPK2	Inhibiting HCV infection	Khatun et al. (2021)
SNHG6	Oncogene	LncSNHG6/MTORC1	Accelerating the progression of NAFLD to HCC	Liu et al. (2022)
H19	Oncogene	AFB1/E2F1/lncRNA H19	Promoting HCC growth and invasion	Lv et al. (2014)
AY927503	Oncogene	LncRNA AY/H1FX	Promoting HCC metastasis	Kang et al. (2019)
LncMER52A	Oncogene	LncMER52A/YY1	Promoting HCC cells migration, invasion, EMT and metastasis	Wu et al. (2020)
ID2-AS1	Tumor suppressor	LncRNA-ID2-AS1/HDAC8/ID2	Inhibiting HCC cells migration, invasion, and metastasis	Zhou et al. (2020)
TCAM1P-004	Tumor suppressor	EZH2/lncRNA TCAM1P-004	Inhibiting HCC cells proliferation	Xu et al. (2019)
RP11-598D14.1	Tumor suppressor	EZH2/RP11-598D14.1/STAU1/PFKFB4	Inhibiting HCC cells proliferation	Xu et al. (2019)
LINC00662	Oncogene	LINC00662/MAT1A/AHCY	Promoting HCC cells proliferation, invasion, and metastasis	Guo et al. (2020)
SNHG6	Oncogene	LncRNA SNHG6/miR-1297/MAT2ALncRNA SNHG6/miR-1297/FUS/MAT1A	Promoting hepatocarcinogenesis	Guo et al. (2018)
GATA3-AS	Oncogene	LncRNA GATA3-AS/KIAA1429/GATA3	Enhancing HCC growth and metastasis	Lan et al. (2019a)
H19	Oncogene	NSUN2/lncRNA H19/G3BP1	Promoting HCC poor differentiation	Sun Z. et al. (2020)
LncMER52A	Oncogene	LncMER52A/p120-catenin/Rac1/Cdc42	Promoting HCC cells invasion and metastasis	Wu et al. (2020)
miR503HG	Tumor suppressor	LncRNA miR503HG/HNRNPA2B1/p52/p65	Inhibiting HCC invasion and metastasis	Wang et al. (2018)
LINC01138	Oncogene	IGF2BP1/IGF2BP3/LINC01138/PRMT5	Promoting HCC proliferation, tumorigenicity, invasion and metastasis	Li et al. (2018)
CASC2	Tumor suppressor	LncRNA CASC2/miR-367/FBXW7	Promoting HCC cells EMT	Wang Y. et al. (2017)
MUF	Oncogene	LncRNA-MUF/ANXA2/miR-34a/Snail1	Promoting HCC cells EMT	Yan et al. (2017)
MCM3AP-AS1	Oncogene	LncRNA MCM3AP-AS1/miR-194-5p/FOXA1	Promting HCC proliferation, cell cycle progression and growth	Wang et al. (2019a)
lnc-Ip53	Oncogene	LncRNA lnc-Ip53/HDAC1/p53/p300	Promoting HCC growth and chemoresistance	Zhang et al. (2020b)
PSTAR	Tumor suppressor	LncRNA PSTAR/HNRNPK/p53	Inhibiting HCC cells proliferation and tumorigenicity	Qin et al. (2020)
TSLNC8	Tumor suppressor	LncRNA TSLNC8/TKT/IL-6/STAT3	Inhibiting HCC invasion and metastasis	Zhang J. et al. (2018)
HOXD-AS1	Oncogene	STAT3/LncRNA HOXD-AS1/miR-130a-3p/SOX4/EZH2/MMP2	Promoting HCC metastasis	Wang H. et al. (2017)
CSMD1-1	Oncogene	LncRNA CSMD1-1/MYC	Promoting HCC cells proliferation, migration, invasion, tumor growth and metastasis	Liu J. et al. (2020)
PXN-AS1-IR3	Oncogene	lncRNA PXN-AS1-IR3/MYC	Promoting HCC metastasis	Zhou H. et al. (2022)
H19	Oncogene	TGF-β/Tgfbr2/Sox2/lncRNA H19	Promoting TIC proliferation, survival and progenitor capacity	Zhang J. et al. (2019)
UTGF	Oncogene	TGF-β/SMAD/lnc-UTGF	Promoting HCC metastasis	Wu et al. (2021)
PTTG3P	Oncogene	LncRNA PTTG3P/PI3K/AKT/PTTG1	Promoting HCC cells proliferation, migration, invasion, tumorigenesis and metastasis	Huang et al. (2018)
CASC11	Oncogene	LncRNA CASC11/PI3K/AKT/mTOR	Promoting HCC proliferation, cell mobility, apoptosis, and cellular metabolism	Song et al. (2020)
T-UCR uc.158-	Oncogene	Wnt/β-catenin/lncRNA T-UCR uc.158-	Promoting HCC growth	Carotenuto et al. (2017)
Lnc-UCID	Oncogene	miR-148a/lnc-UCID/DHX9/CDK6	Promoting HCC cells G1/S transition and growth	Wang T. et al. (2019)
Lnc-APUE	Oncogene	Lnc-APUE/HNF4α/miR-20b/E2F1	Promoting HCC cells G1/S transition and growth	Li et al. (2021)
PINT87aa	Tumor suppressor	LncRNA PINT87aa/FOXM1/PHB2	Inducing HCC cells senescence	Xiang et al. (2021)
HULC	Oncogene	LncRNA HULC/miR15a/P62	Promoting HCC cells growth and autophagy	Xin et al. (2018)
MALAT1	Oncogene	BA/lncRNA MALAT1/miR-22-3p/IAP	Inhibiting HCC growth, induceing HCC cells death and apoptosis	Chen F. et al. (2020)
NEAT1	Tumor suppressor	p53/lncRNA NEAT1/miR-362-3p/MIOX	Increasing the anti-tumor activity of erastin and RSL3	Zhang Y. et al. (2022)
NEAT1_2	Tumor suppressor	MTORC1/lncRNA NEAT1_2/NONO/SFPQ	Restraining AKT-mTORC1-mediated aerobic glycolysis and liver tumor development	Zhang H. et al. (2022)
MALAT1	Oncogene	mTORC1/lncRNA MALAT1/TCF7L2	Regulating HCC glucose metabolism, enhancing glycolysis, and inhibiting gluconeogenesis	Malakar et al. (2019)
WFDC21P	Tumor suppressor	Nur77/lncRNA WFDC21P/PFKP/PKM2	Inhibiting HCC proliferation, growth, metastasis	Guan et al. (2020)
LINC01554	Tumor suppressor	LINC01554/PKM2/Akt/mTOR	Impairing HCC cells aerobic glycolysis	Zheng et al. (2019)
HNF1A-AS1	Tumor suppressor	HNF1α/lncRNA HNF1A-AS1/SHP-1	Inhibiting HCC tumorigenesis and metastasis	Ding et al. (2018)
NEAT1	Oncogene	LncRNA NEAT1/miR-124-3p/ATGL/DAG/FFA	Promoting HCC cells growth, disrupting the lipolysis of HCC cells	Liu et al. (2018)
PRLH1	Oncogene	p53/lncRNA PRLH1/RNF169	Promoting HCC cells proliferation and the HR-mediated DNA repair	Deng et al. (2019)
NIHCOLE	Oncogene	LncRNA NIHCOLE	Promoting HCC cells ligation efficiency of DNA double-strand breaks	Unfried et al. (2021)
HAND2-AS1	Oncogene	LncRNA HAND-AS1/INO80/BMPR1A	Promoting HCC stem cells self-renewal	Wang et al. (2019b)
lncRNA-MUF	Oncogene	LncRNA-MUF/ANXA2/Wnt/β-catenin	Promoting HCC EMT	Yan et al. (2017)
LINC01133	Oncogene	LINC01133/ANXA2/STAT3/cyclinD1	Promoting HCC aggressive and EMT	Yin et al. (2021)
SNHG10	Oncogene	LncRNA SNHG10/miR-150-5p/c-Myb/RPL4/SOX9	Promoting HCC cells proliferation, invasion, migration, cell cycle and EMT	Lan et al. (2019b)
HCCL5	Oncogene	TGF-β1/lncRNA HCCL5/ZEB1	Promoting HCC cells viability, migration, and EMT	Peng et al. (2019)
HOXD-AS1	Oncogene	LncRNA HOXD-AS1/miR19a/ARHGAP11A/RGS3/MEK/ERK	Promoting HCC metastasis and growth, Inhibiting Dox-induced apoptosis	Lu et al. (2017)
LINC01278	Oncogene	Wnt/β-catenin/LINC01278/TCF-4/miR-1258/Smad2/3	Promoting HCC cells migration, invasion and metastasis	Huang W. J. et al. (2020)

## The relationship between lncRNAs and risk factors of hepatocellular carcinoma

### Hepatitis B virus infection

The hepatitis B virus (HBV) infection is linked to an elevated risk of developing HCC (Hepatitis, 2018). HBV can be identified as a possible cause of HCC if the replication of viral DNA is detected. The viral HBV X protein (HBx) has been shown to induce host gene expression and promote HCC progression (Kim et al., 1991; Hepatitis, 2018). Patients with HBV infection remain in a chronic condition because HBx regulates the transcription of the HBV gene template covalently closed circular DNA (cccDNA), which in turn modifies the chromatin structure of the virus (Protzer, 2015; Wei and Ploss, 2020; Wei and Ploss, 2021).

**FIGURE 1:**
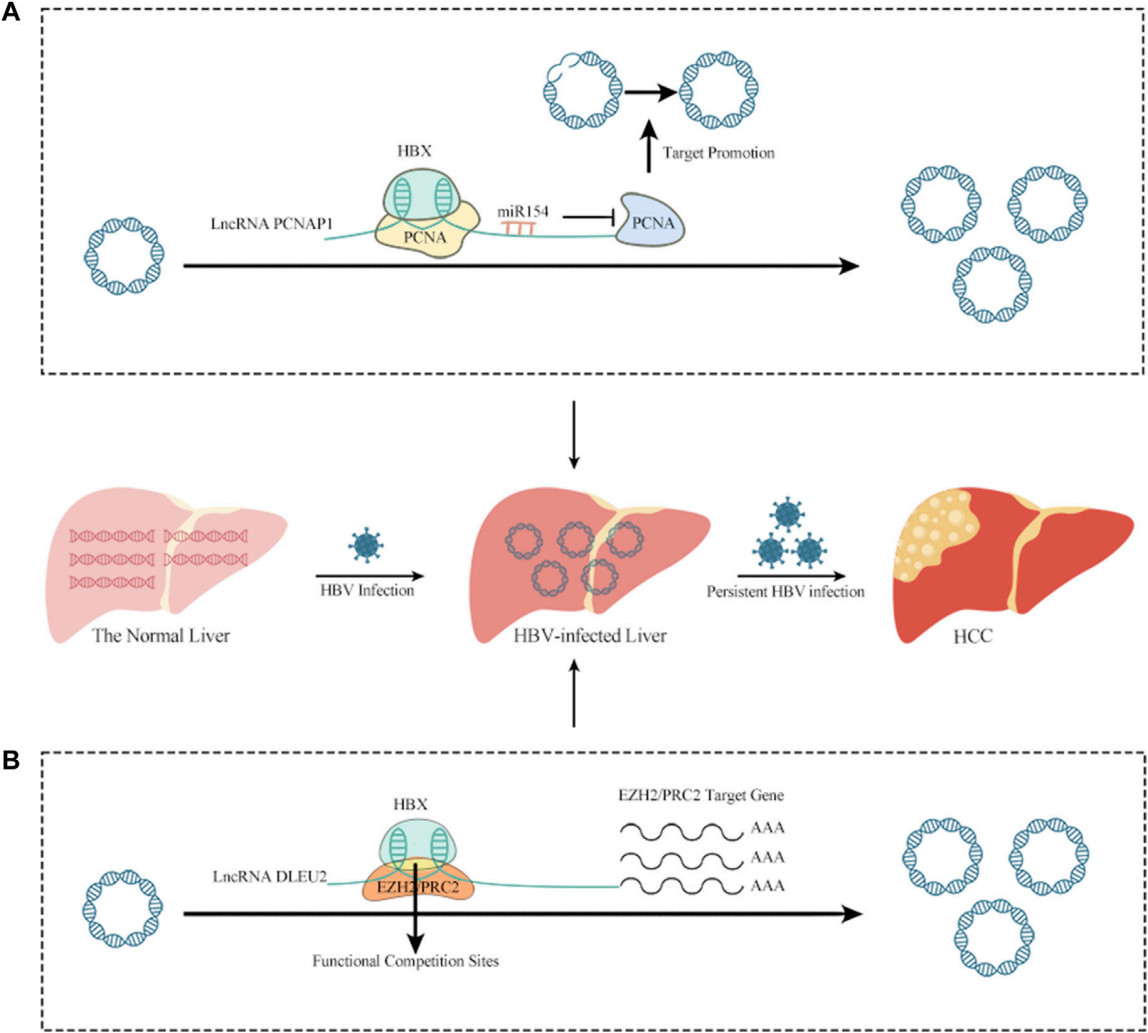
The relationship between lncRNAs and HBV infection of HCC. **(A)** PCNAP1 sponges miR-154, promoting expression of hepatic PCNA, which is required for cccDNA formation and is involved in DNA replication, repair, RNA modification, and chromatin remodeling. PCNA then binds to HBc, enabling anchorage to the cccDNA mini-chromosome and subsequent vital replication and cccDNA accumulation. **(B)** In HBV-infected cells, HBx binds to the lncRNA DLEU2 promoter, promoting DLEU2 transcription and its subsequent accumulation in infected hepatocytes. Blocking the repressive function of EZH2/PRC2 and activating transcription leads to the transcription and replication of cccDNA in HBV-related HCC, together with the transcriptional activation of genes downstream of EZH2/PRC2 and, ultimately leading to HCC.

LncRNAs have been linked to HCC caused by HBV infection (Zhang et al., 2014; Wang et al., 2021). The lncRNA DLEU2 is induced to be transcribed by HBx in HBV-infected cells, leading to an increase in DLEU2 levels in infected hepatocytes. In HBV-related HCC, the transcription and replication of cccDNA, together with the transcriptional activation of genes downstream of Enhancer of Zeste Homolog 2/Polycomb Repressive Complex 2 (EZH2/PRC2), leads to HCC *via* a cascade of events. This suggests that the lncRNA DLEU2-HBx interaction may be useful as a target for treating HBV-related HCC (Salerno et al., 2020). PCNAP1, another lncRNA, also induces HBV replication and resultant increases in cccDNA. This occurs because PCNAP1 sponges miR-154, promoting expression of hepatic Proliferating cell nuclear antigen (PCNA), which is required for cccDNA formation and is involved in DNA replication, repair, RNA modification, and chromatin remodeling. PCNA then binds to HBc, enabling anchorage to the cccDNA mini-chromosome and subsequent vital replication and cccDNA accumulation (Kelman, 1997; Feng et al., 2019; Wei and Ploss, 2020; Wei and Ploss, 2021) (Figure 1). A lncRNA variation was shown to increase HCC risk but aid in HBV remission *via* a genome-wide association analysis. The authors identified an rs2844512G>C variant in LINC01149, which enabled LINC01149 to sponge miR128-3p and lower its expression, leading to increased levels of MHC class-I chain-related gene A (MICA). On the one hand, MICA promotes HBVSR (hepatitis B virus spontaneous recovery), which benefits from the efficient activation of NK cell recognition and cytotoxicity by the interaction of MICA with NKG2D on NK cells, resulting in the lysis of infected hepatocytes. On the other hand, MICA increases the risk of HCC, as LINC01149 contributes to increased soluble MICA (sMICA) levels by targeting specific alleles. Studies have shown that higher levels of sMICA can promote NK cell depletion and tumor immune escape, which may be related to the fact that up-regulated MICA can support persistent HBV infection, leading to increased susceptibility to HCC (Groh et al., 2002; El-Gazzar et al., 2013; Tong et al., 2013; Zhong et al., 2020).

### Hepatitis C virus infection

Chronic hepatitis C (CHC) is the result of an hepatitis C virus (HCV) infection. Obesity, insulin resistance, and hepatic steatosis can further drive the progression of CHC to HCC, influenced by the host’s metabolic state. However, in late CHC, liver problems such as ageing, inflammation, and post-sustained viral response (SVR) metabolic dysfunction are risk factors for HCC, although direct-acting antiviral (DAA) has dramatically increased the cure rate of CHC (Reig et al., 2016; Innes et al., 2018; Nishibatake Kinoshita et al., 2019; Leslie et al., 2022).

HCV disrupts gene expression in hepatocytes, seen in hypomethylation of whole-genome DNA and the dysregulation of enhancer expression. Even if HCV is cured, these abnormal epigenetic and transcriptional changes are not restored, resulting in a persistently elevated risk of HCV-induced HCC (Hlady et al., 2022). LncRNAs also regulate gene expression in HCV-infected hepatocytes. For instance, lncRNA-IFI6 mediated modification of histones stimulates HCV replication by preventing IFI6 transcription (Liu et al., 2019) and the levels of lncRNA Linc-Pint are reduced in hepatocytes infected with HCV. Linc-Pint binds to Serine-arginine protein kinase 2 (SRPK2), reducing the expression of the latter and inhibiting *de novo* lipogenesis, thus blocking HCV infection (Khatun et al., 2021).

### Other risk factors

Many studies have shown that not only obesity, smoking, and diabetes mellitus type 2 (Dhanasekaran and Felsher, 2019; Fujii et al., 2020; Premkumar and Anand, 2021; Qi et al., 2021) but also nutritional factors are risk important factors for HCC (Ruiz-Margáin et al., 2021). Non-alcoholic fatty liver disease (NAFLD) is a potential outcome of malnutrition. Some studies have shown that the lncRNA SNHG6 may promote the transition from NAFLD to HCC, possibly through activation of cholesterol-induced mechanistic target of rapamycin complex 1(MTORC1) (Campisano et al., 2019; Liu et al., 2022). However, nutritional imbalance, including high fructose or high sugar intake, can also stimulate hepatic steatosis as well as induce hepatic fibrosis through the gut-liver axis, potentially leading to HCC (Febbraio and Karin, 2021). However, some plant foods, such as potatoes, carrots, apples, and papaya, are reported to prevent HCC (Zhang D. et al., 2020; Saeed et al., 2022).

Excess alcohol consumption has been reported to disrupt the gut flora and translocate into the bloodstream, contributing to liver damage (Meroni et al., 2019; Moreno-Gonzalez and Beraza, 2021). Moreover, alcohol can synergize with HBV/HCV infection to promote the metabolic reprogramming of hepatocytes/liver tumor initiating cells (TICs), initiating their self-renewal and potentially leading to HCC (Machida, 2020). Dysregulation of lncRNAs has been observed in alcohol-induced HCC (Zheng et al., 2018) although the mechanism by which they cooperate with alcohol-induced HCC remains to be studied.

It is well documented that aflatoxin B1 (AFB1) causes HCC. AFB1 interacts synergistically with HBV/HCV to promote HCC development through epigenetic modifications (Ferreira et al., 2019; McCullough and Lloyd, 2019; Rushing and Selim, 2019). Studies have found that AFB1 exposure can exert immunosuppressive properties through lncRNAs (Ferreira et al., 2019; Chao et al., 2022). AFB1 interacts with E2 promoter binding factor 1 (E2F1) and upregulates lncRNA H19 levels, promoting HCC growth and metastasis (Lv et al., 2014). Further, it was found that AFB1 may induce the up-regulation of anti-apoptotic lncRNA expression to promote increased AFB1 carcinogenesis (Shi et al., 2016; Liu X. et al., 2020).

## LncRNAs and gene expression in hepatocellular carcinoma

The functional structure of chromatin is the basis of epigenetics. Chromatin modifications, including histone modifications and DNA methylation, are important “languages” for regulating gene expression, thus potentially modulating the entire structure and functions of the cell. However, numerous studies have shown that dysregulation of these chromatin modification languages can cause aberrant gene expression, which is one of the key causes of many malignancies (Zhao et al., 2021).

### Chromatin modification

LncRNAs are also implicated in epigenetic modifications of chromatin in HCC (Marrero et al., 2005). LncRNA AY927503 (AY) is strongly expressed in HCC, where it promotes metastasis through regulation of integrin subunit alpha-V gene (ITGAV) expression. The underlying mechanism is that AY interacts with histone 1 FX (H1FX), leading to the recruitment of epigenetically modified histone H3 lysine 4 trimethylation (H3K4me3) and histone H3-lysine 9-lysine 14 acetylation (acH3K9/14) to the promoter region of integrin αV (ITGAV), displacing tri-methylation at lysine 27 of histone H3 (H3K27me3) and H1FX. This, in turn, leads to chromatin remodeling through the actions of RNA polymerase II to promote cancer progression (Kang et al., 2019). LncMER52A is only expressed in HCC cells, and its activation is regulated both by chromatin modification and the transcription factor yin-yang 1 (YY1). High levels of lncMER52A are associated with significantly increases in H3K4me3 and H3K27ac. Furthermore, YY1 binds the lncMER52A promoter to activate lncMER52A transcription. These epigenetic mechanisms promote HCC tumorigenesis (Wu et al., 2020). LncRNA-ID2-AS1, on the other hand, acts as a tumor suppressor, preventing HCC migration and metastasis. Mechanistically, by controlling the binding of H3K27ac to the ID2 promoter and RNA polymerase II (RNAPII) to the ID2 enhancer domain, ID2-AS1 acts as a chromatin modifier to regulate ID2 transcription. This results in epigenetic modification and activation of the enhancer and modulation of ID2 expression which, in turn, regulates Epithelial-Mesenchymal Transition (EMT) progression to suppress HCC metastasis (Zhou et al., 2020).

Two lncRNAs, TCAM1P-004 and RP11-598D14.1, were discovered as tumor suppressors in HCC by a genome-wide screen and functional analysis. EZH2, a major component of the Polycomb repressive complex 2 (PRC2), acts as a histone methyltransferase and hence silences the expression of tumor suppressor genes. Transcriptional activity for TCAM1P-004 and RP11-598D14.1 is suppressed as a result of EZH2 catalyzing the production of H3K27me3. Additionally, the study authors discovered that RP11-598D14.1 interacts with the RNA-binding protein Staufen1 (STAU1) to suppress the expression of the oncogene phosphofructo-2-kinase/fructose-2,6-biphosphatase 4 (PFKFB4). RP11-598D14.1 expression is low in HCC, potentially leading to increased levels of PFKFB4 which, in turn, may lead to HCC (Xu et al., 2019).

### Methylation modification of genes

The hypomethylation status of the genome has been linked to HCC (Meunier et al., 2021). High levels of LINC00662 are seen in HCC, associated with downregulation of methionine adenosyltransferase 1A (MAT1A) and S-adenosylhomocysteine hydrolase (AHCY). This reduces the levels of S-adenosylmethionine (SAM) and increases those of S-adenosylhomocysteine (SAH) levels, altering methylation of the LINC00662 promoter and, in turn, leading to hypomethylation of the genome. Upregulation of MAT1A and AHCY contributes to antitumor effects (Guo et al., 2020). The lncRNA SNHG6 acts as a “molecular switch” to modulate intracellular S-adenosylmethionine (SAMe) concentrations through two coupled positive feedback loops. First, SNHG6 acts as a ceRNA to inhibit miR-1297 expression, thus increasing methionine adenosyltransferase 2A (MAT2A) levels. Second, SNHG6 stabilizes fused in sarcoma (FUS) mRNA by reducing its miR-1297-induced decay. Then, FUS binds to MAT1A mRNA and hinders nuclear export of MAT1A mRNA, thereby reducing MAT1A protein synthesis. It can be concluded that the reduction of MAT1A and the increase of MAT2A, two key enzymes of SAMe, can suppress SAMe levels which, in turn, leads to genome-wide hypomethylation. Further, it has been found that exogenous SAMe can inhibit the effect of SNHG6 on genome-wide hypomethylation, which provides new evidence for tumor methylation therapy (Guo et al., 2018).

### RNA modification

More than 100 chemical modifications of RNA have been shown over the past 50 years using a variety of methods. These highly modified forms of covalent binding have been extensively characterized, with the best documented including N6-methyladenosine (m6A), 5-methylcytosine (m5C), N1-methyladenosine (m1A), internal 7-methylguanosine (m7G)), RNA cap methylations, pseudouridine, and uridylation (Barbieri and Kouzarides, 2020). RNA modification plays a significant part in cancer development, and proteins associated with the process can serve as potential anticancer targets (Delaunay and Frye, 2019; Boriack-Sjodin et al., 2022).

The lncRNA GATA3-AS serves as a guide RNA to bind KIAA1429 to GATA binding protein 3 (GATA3) pre-mRNA, and KIAA1429 is a component of the m6A methyltransferase complex. Increased m6A methylation of the 3′UTR of GATA3 pre-mRNA is induced in HCC cells after KIAA1429 targeting. As a result, GATA3 expression is downregulated, cancer and metastasis are promoted, and normal hepatocytes lose their stability as a result of the HuR-GATA3 interaction (Shi et al., 2020). Catalyzing m5C RNA modifications, NSUN2 is an RNA methyltransferase. Many studies have shown that lncRNA H19 is carcinogenic, and it has been suggested that this is related to its RNA modification. Sun et al. demonstrated that in HCC cells, NSUN2 increased the methylation level of H19, which was found to be positively correlated with poor HCC differentiation. Moreover, the oncoprotein GTPase-activating protein SH3 domain-binding protein 1 (G3BP1) can specifically bind to the m5C-modified lncRNA H19, a further indication of the part played by the m5C modification of H19 lncRNA in HCC malignancy (Sun Z. et al., 2020). Other related RNA modifications of lncRNAs in HCC remain to be studied (Shi et al., 2020; Luo et al., 2022).

### Ubiquitination-associated degradation

Ubiquitination-deubiquitination is a post-translational modification associated with protein degradation *via* the ubiquitin-proteasomal pathway. Dysregulation of ubiquitination can lead to cancer development and there have been numerous investigations into the targeting of ubiquitin-proteasomal pathway for cancer treatment. However, these efforts have not proved entirely successful due to drug resistance and research is now being directed into the identification of suitable biomarkers to expand the treatment strategy. LncRNAs are involved in the cellular ubiquitination degradation pathway in HCC, and may represent feasible therapeutic targets (Narayanan et al., 2020; Sun T. et al., 2020; Cockram et al., 2021; Dang et al., 2021).

High expression of lncMER52A is related to poor HCC prognosis. LncMER52A promotes HCC tumorigenesis through modulation of p120-catenin/Rac1/Cdc42 signaling. LncMER52A inhibits the ubiquitin/proteasome-dependent degradation of p120-catenin, thus promoting its stability, as well as regulating the activity of downstream GTPases leading to its oncogenic effect (Wu et al., 2020). LncRNA miR503HG levels are reduced in HCC and have been linked to poor prognosis, suggesting its potential as a biomarker. Interacting with heterogeneous nuclear ribonucleoprotein A2/B1 (HNRNPA2B1) and so inhibiting NF-kB signaling has been demonstrated to prevent HCC metastasis. Inhibition of the NF-kB signaling pathway and suppression of metastasis are the results of LncRNA miR503HG’s role in lowering HNRNPA2B1 protein levels through increased ubiquitination and degradation of the protein, as well as *via* the degradation of the p52 and p65 mRNAs that influence NF-kB signaling (Wang et al., 2018). The LncRNA LINC01138 was shown to be upregulated in HCC, and studies proved its oncogene status in this disease. IGF2BP1/IGF2BP3 stabilizes LINC01138 and, in HCC, LINC01138 binds specifically to protein arginine methyltransferase 5 (PRMT5), preventing its ubiquitination and degradation and enabling its oncogenic function. Several PRMT5 inhibitors have been used in cancer therapy, indicating that the study of the carcinogenic mechanisms of lncRNAs will further our understanding of HCC, allowing the development of new drugs targeting its pathways and effective prediction of patient prognosis (Li et al., 2018).

### Competing endogenous RNA mechanism

**FIGURE 2:**
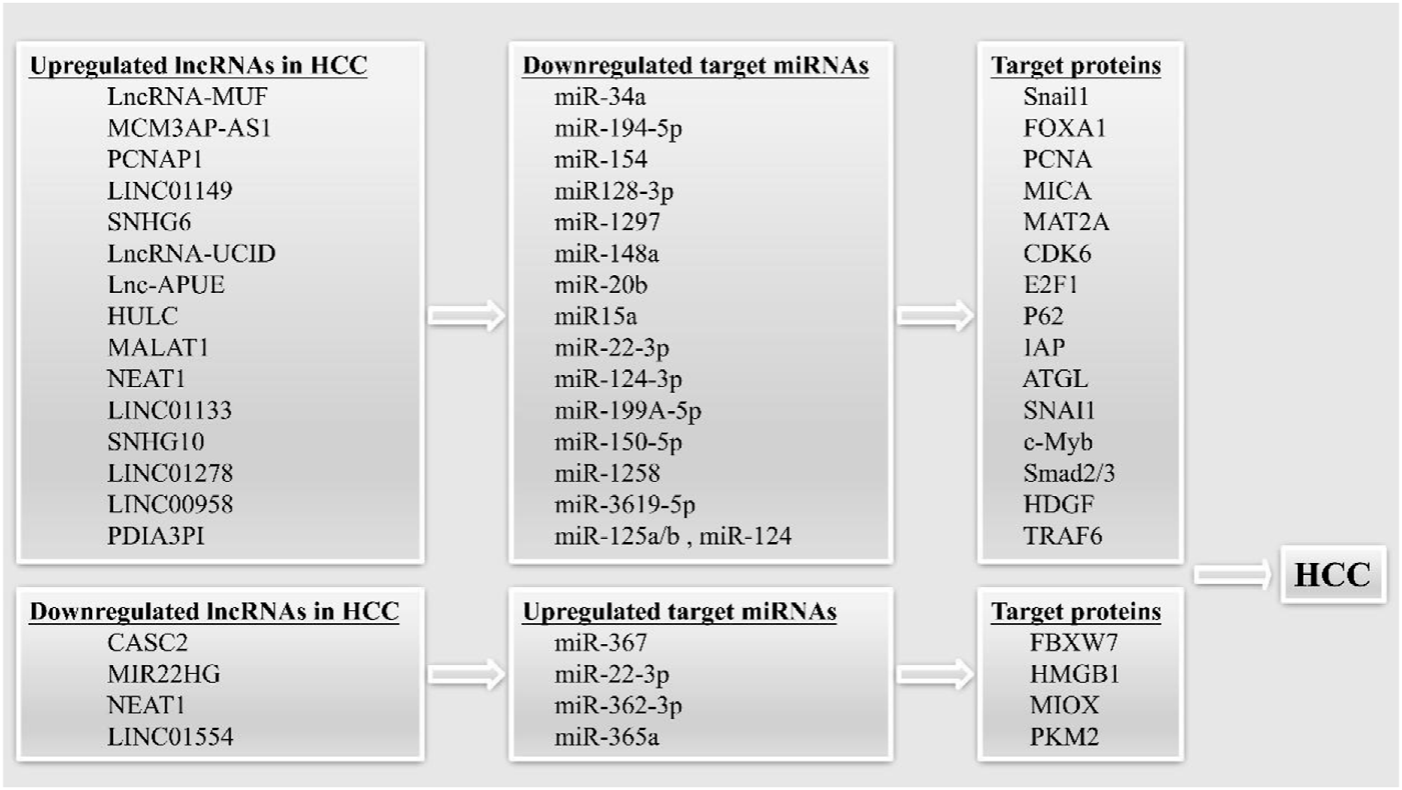
LncRNAs can act as competing endogenous RNAs (ceRNAs) in HCC. The upregulated and downregulated lncRNAs can sponging target miRNAs, leading to increased or decreased expression of target proteins, thereby promoting the occurrence of HCC.

It is been shown that lncRNAs may play a role in carcinogenesis as ceRNAs (competing endogenous RNAs) (Figure 2). Successful biomarkers may now be investigated with the use of ceRNAs, due to the advancement of ceRNA network research (Chan and Tay, 2018; Qi et al., 2020).

The tumor suppressor lncRNA CASC2 functions as a ceRNA, decreasing miR-367 levels and causing an increase in F-box with 7 tandem WD40 (FBXW7) levels, which in turn inhibits EMT and invasion and migration in HCC cells (Wang Y. et al., 2017). LncRNA-MUF has been shown to have high expression in HCC and to be associated with a dismal prognosis in this disease. By acting as a ceRNA, the LncRNA-MUF suppresses the tumor-suppressing function of miR-34A, which in turn increases Snail1 expression and promotes EMT (Yan et al., 2017). Researchers have also identified a tumor suppressor lncRNA, MIR22HG, which is downregulated in HCC. On the one hand, MIR22HG inhibited the expression of HMGB1 through miR-22-3p, inhibiting high mobility group box-1 (HMGB1) signaling to reduce HCC cell proliferation and metastasis. On the other hand, MIR22HG was found to compete with human antigen R (HuR)-targeted oncogenes for binding to HuR, thus destabilizing the oncogenes and reducing HCC progression (Zhang D. Y. et al., 2018). MCM3AP-AS1 was identified by Wang et al. as a lncRNA that served as a ceRNA and bound miR-194-5p to induce forkhead box protein A1 (FOXA1) overexpression and HCC (Wang et al., 2019b).

## The relationships between lncRNAs and signaling pathways in hepatocellular carcinoma

The involvement of lncRNAs in cancer-associated signaling pathways is well-documented. It is, thus, important to investigate these pathways to identify potential targets for drug development (Peng et al., 2017; Lin and Yang, 2018).

### p53 signaling

The acetylation of p53 is crucial for its function as a tumor suppressor (Wang et al., 2016). When p53 is activated, it binds directly to the p53-responsive element (p53RE) in the lncRNA lnc-Ip53 promoter, inducing lnc-Ip53 transcription. As a result, Lnc-Ip53 interacts with histone deacetylase 1 gene (HDAC1) to inhibit its ubiquitination, which in turn increases HDAC1 levels and consequently p53 deacetylation. To prevent the acetylation of p53 by p300, Lnc-Ip53 also competes with p53 for binding to p300. These findings indicate that p53/lnc-Ip53 forms a negative feedback loop that inhibits p53 acetylation/activity and thus promotes both HCC development and chemoresistance (Zhang et al., 2020b). Increased p53 signaling and HCC suppression are the results of lncRNA PSTAR’s regulation of p53 activity by preventing de-SUMOylation of heterogeneous nuclear ribonucleoprotein K (Qin et al., 2020).

### STAT3 signaling

Reduced expression of the tumor suppressor LncRNA TSLNC8 is associated with a poor prognosis in HCC. Several kinds of tumors show persistent activation and high levels of signal transducer and activator of transcription 3 (STAT3) (Yu et al., 2009; He and Karin, 2011; Zou et al., 2020). Interactions between TSLNC8 and transketolase (TKT) and STAT3 suppress phosphorylation and transcription of STAT3, hence inactivating the IL-6/STAT3 pathway and lowering tumorigenesis. TSLNC8 inhibits phosphorylation of STAT3 at tyrosine 705, or Y705, while increasing phosphorylation of serine 727, or S727. This results in increased levels of phosphorylated STAT3-S727 and decreased levels of phosphorylated STAT3-Y705 in the nucleus. Because of this change, STAT3’s phosphorylation state and nuclear localization are both modified, which in turn affects the protein’s biological function. The authors further found that TKT and STAT3 phosphorylation at either Tyr705 or Ser727 can independently predict HCC prognosis. Interestingly, TSLNC8 may prevent the interaction of TKT and STAT3. Overexpression of TSLNC8 blocks interactions between TKT and STAT3, suggesting competition between TSLNC8 and TKT or STAT3, affecting STAT3 phosphorylation. IL-6 is known to activate STAT3, enhancing phosphorylation at Tyr705 and reducing phosphorylation at Ser727. In addition, as a result of its effect on the IL-6-STAT3 pathway, TSLNC8 is also able to inhibit carcinogenesis in HCC. Therefore, the TSLNC8-TKT/IL-6-STAT3 axis shows promise as a therapeutic target for HCC (Zhang J. et al., 2018). LncRNA HOXD-AS1, which is controlled by STAT3 and increased in HCC, was discovered to increase SRY-related high-mobility-group box 4 (SOX4) expression by binding competitively to miR-130a-3p and activating two direct SOX4 targets, EZH2 and matrix metallopeptidase 2 (MMP2). This resulted in the promotion of HCC metastasis (Wang H. et al., 2017).

### MYC signaling

LncRNA CSMD1-1 is upregulated in HCC, where it bind specifically to MYC to inhibit its ubiquitination and degradation, thereby enhancing MYC signaling to promote HCC progression (Liu J. et al., 2020). LncRNA PXN-AS1-IR3 is recently identified abnormally spliced isoform produced by DDX17-induced intron retention in the third intron of lncRNA-PXN-AS1. DDX17 is upregulated in HCC where it induces MYC transcriptional activation by inducing PXN-AS1-IR3 production which, in turn, promotes HCC metastasis (Zhou H. et al., 2022).

### TGF-β signaling

Transforming growth factor-β (TGF-β) has both pro- and anticancer effects in HCC pathogenesis (Majumdar et al., 2012). An “early TGF-β signature” has been described (Coulouarn et al., 2008), characterized by lower tumor stage, reduced serum AFP, and improved patient survival, indicative of tumor suppression by TGF-β. TGFBR2 incactivation in tumor-initiating hepatocytes (TICs) was found to promote HCC progression, also suggesting a tumor-suppression role for TGF-β in TICs. Notably, deletion of transforming growth factor-β receptors 2 (TGFBR2) elevated levels of lncRNA H19, similar to the effects of SRY-related high-mobility-group box 2 (SOX2) overexpression. As SOX2 functions as a transcription factor in promoting the cancer stem cell phenotype, this suggests that the tumor-suppressing effects of TGF-β are associated with SOX2 inhibition of lncRNA H19 transcription in TICs (Zhang J. et al., 2019). Furthermore, a positive feedback loop involving TGF-β/SMAD/lnc-UTGF has been discovered, which stimulates HCC metastasis. When TGF-β is present, it promotes SMAD2/3 binding to the lnc-UTGF promoter, which in turn stimulates transcription of lnc-UTGF, which then interacts with and stabilizes SMAD2/4 mRNA. HCC metastasis is facilitated by the loop because of its enhancement of TGF-β signaling (Wu et al., 2021).

### PI3K/AKT signaling

LncRNA PTTG3P levels are raised in HCC in association with pituitary tumor-transforming gene 1 (PTTG1) expression. Pituitary tumour-transforming 3, pseudogene (PTTG3P) activates the PI3K/AKT pathway, and elevated levels of PTTG1 and PI3K/AKT pathway activation induce the G1/S phase transition in HCC cells, increasing proliferation and promoting the EMT (Huang et al., 2018). YY1 is reported to target the lncRNA CASC11 promoter, leading to increased expression of the lncRNA in HCC. CASC11 also upregulates E2F1 expression through recruitment of eukaryotic translation initiation factor 4A3 (EIF4A3), activating the NF-κB and PI3K/AKT/mTOR pathways to promote HCC progression. Programmed cell death-Ligand 1 (PD-L1) levels were also reduced after CASC11 inhibition, suggesting that CASC11-mediated HCC progression is linked to PD-L1 induction of immune escape (Song et al., 2020).

### Wnt signaling

Investigation of the T-UCR downstream of the Wnt/β-catenin pathway in HCC identified a lncRNA T-UCR uc.158-. T-UCR activation through Wnt signaling may promote HCC development and progression. It was shown that uc.158- was exclusively turned on in cancer cells, indicating a specialized involvement in Wnt signaling and a possible therapeutic target (Carotenuto et al., 2017).

## Relationships between lncRNAs and hepatocellular carcinoma cell cycle regulation

Malignant proliferation is a hallmark of malignant tumors. Cell cycle dysregulation contributes to this prolonged proliferation (Matthews et al., 2022). Several proteins, such as cyclin-dependent kinases (CDK4/6) are responsible for controlling the G1/S phase transition, which is crucial in determining cell growth or arrest (Choi and Anders, 2014). In cancer cells, the normal regulation of the cell cycle is dysregulated, leading to uncontrolled cell division (Matthews et al., 2022). Cancer development and progression are mediated by regulatory networks associated with lncRNAs, as pointed out by Guiducci and Stojic (2021).

### Cell cycle

The 850–1030 nt domain of Lnc-UCID has been found to have a role in cell cycle progression by binding competitively to DExH-Box helicase 9 (DHX9) and lowering DHX9’s binding to the 3′UTR of CDK6 mRNA. Proliferation was stimulated because CDK6 levels were raised, the G1/S transition was favored, and post-transcriptional regulation of CDK6 expression was blocked. In addition, miR-148A could bind directly to lnc-UCID resulting in its downregulation, suggesting that increased levels of lnc-UCID in HCC may be the consequence of either its amplification or reduced expression of miR-148a (Wang et al., 2019a). Furthermore, lnc-APUE, which is elevated in HCC and associated with patients’ risk of recurrence-free survival (RFS), has been shown to have a role in the cell cycle. The G1/S transition and tumor development are also stimulated by Lnc-APUE. Hepatocyte nuclear factor 4α (HNF4α) binds the lnc-APUE promoter to reduce lnc-APUE transcription, which in turn decreases E2F1 expression and cell proliferation, consistent with reports that E2F1 regulates the cell cycle. In HCC, lnc-APUE can sponge miR-20b, thereby preventing miR-20b-mediated repression of E2F1, leading to increased levels of E2F1 expression and levels and augmented G1/S transition (Li et al., 2021).

### Cellular senescence and death

Excessive stress can result in an arrest of cell proliferation, a phenomenon known as cellular senescence. Harnessing cellular senescence has emerged as a promising modality for antitumor therapy (Wang et al., 2022). The induction of cancer cell senescence is a key step in an anti-tumor program that leads to cell cycle arrest, which prevents cancer progression by limiting cancer cell proliferation. However, senescent cells do not lose their functionality permanently or completely. According to the available data, senescent cancer cells may re-enter the cell cycle and reestablish cancer stemness, which can result in tumor recurrence. Therefore, anti-aging therapy, or the elimination of senescent cancer cells, may boost the efficacy of cancer ageing treatment and reduce the likelihood of tumor recurrence. The challenge facing current cancer aging treatment is mainly a lack of biomarkers for accurate detection of the aging status of the cancer cells. Using drugs to trigger senescence in different cancer cell lines, Jochems et al. established a SENCAN classifier aimed at identifying senescence-specific genes. Several genes with lncRNA signatures were included in the SENCAN classifier, indicating their involvement in cancer cell senescence (Muñoz-Espín and Serrano, 2014; Jochems et al., 2021).

HCC senescent cells produce the LncRNA PINT87aa, which has been demonstrated to promote senescence and suppress proliferation in HCC cells. PINT87aa binds to forkhead box M1 (FOXM1) to inhibit prohibitin 2 (PHB2) transcription, further inhibiting PHB2-mediated mitophagy, blocking the cell cycle, and inducing cellular senescence (Xiang et al., 2021).

Autophagy is important in tumorigenesis (White et al., 2015), and lncRNA HULC has been shown to be involved in autophagy in HCC cells. However, lncRNA HULC is highly elevated in HCC, and its promotion of autophagy is reflected in increased expression of Beclin 1 and the autophagy marker LC3II. By encouraging autophagy, phosphatase and tensin homolog (PTEN) is more effectively ubiquitinated and degraded. However, by decreasing miR15a levels, HULC increases the production of p62, which is essential for PTEN’s autophagic breakdown. This suggests that HULC reduces PTEN through the autophagy-p62-mediated ubiquitin-proteasomal system, thereby promoting the development of HCC (Xin et al., 2018). Betulinic acid (BA) has been shown in several studies to induce cell death in various cancers. In HCC, BA was shown to block the carcinogenic lncRNA MALAT1 by lowering its levels. This in turn promoted apoptosis of HCC cells by targeting inhibitor of apoptosis (IAP) through miR-22-3p, therefore preventing HCC from progressing. BA was also found to induce autophagic flux in HCC cells, resulting, ultimately, in cell death, with IAPs likely to be key to this regulatory process (Chen F. et al., 2020).

Programmed cell death known as ferroptosis has just lately been identified (Mou et al., 2019). According to the literature, the lncRNA NEAT1 induces ferroptosis in HCC cells. After subjecting cells to ferroptosis inducers erastin and RAS-selective lethal (RSL3), the scientists discovered p53 upregulated NEAT1 levels. Ferroptosis elicited by erastin and RSL3 is facilitated by NEAT1 sponging miR-362-3p to increase myo-Inositol oxygenase (MIOX) levels. There is evidence that ferroptosis inducers enhance cellular sensitivity to chemotherapy (Liang et al., 2019). Since lncRNAs seem to play important roles in HCC ferroptosis, NEAT1 may be a viable therapeutic target when used in conjunction with treatments that specifically target ferroptosis (Zhang Y. et al., 2022).

### Cellular metabolism

Malignancy is also characterized by the resetting of cellular metabolism. Cancer cells promote tumor growth *via* the use of internal mechanisms that drive metabolic alterations and interactions with cytokines in the tumor microenvironment (TME) (Dey et al., 2021; Martínez-Reyes and Chandel, 2021).

Cancer cells typically use glycolysis to maintain their metabolic energy and promote tumor growth, termed the “Warburg effect” (Warburg, 1925). The “Warburg effect” is characteristic of cancer and involves the promotion of cancer growth by switching glucose production from oxidative phosphorylation to aerobic glycolysis through the MTORC1 pathway (Szwed et al., 2021). It is documented that HCC is closely related to aerobic glycolysis. MTORC1 inhibits lncRNA NEAT1_2 activation in HCC, and NEAT1_2 is essential for paraspeckles, constituting its “architectural backbone.” Additionally, it can sequester NONO and SFPQ, both RNA-binding proteins (Yamazaki et al., 2018). Reductions in NEAT1_2 levels lead to the release of NONO/SFPQ, allowing NONO to bind to specific motifs in key glycolytic enzymes. This regulates pre-mRNA splicing of these enzymes leading to the promotion of aerobic glycolysis for supplying energy. This binding can be inhibited by rapamycin. Rapamycin inhibits HCC cell growth under high-glucose conditions, and NEAT1_2 knockdown attenuates this inhibition, further suggesting the effects of rapamycin on HCC glucose metabolism-related processes, possibly through inhibition of the mTORC1 -NEAT1_2 axis (Zhang H. et al., 2022). MALAT1, a lnRcNA, is also linked to the Warburg effect. Because of mTORC1’s stimulation of cap-dependent translation, MALAT1 stimulates the translation of the transcription factor TCF7L2, which in turn increases the expression of glycolytic genes and negatively regulates gluconeogenesis to preserve the oncogenic features of HCC cells. Cancer glucose metabolism may benefit from a novel approach including the suppression of TCFL2 or downregulation of the lncRNA MALAT1 (Malakar et al., 2019). LncRNA WFDC21P inhibits HCC tumorigenesis in both cell lines and *in vivo* through Nur77 enhancement of WFDC21P expression. The interaction of WFDC21P with two key glycolytic enzymes, phosphofructokinase-P (PFKP) and pyruvate kinase M2 (PKM2), is enhanced by WFDC21P overexpression. Inhibiting PFKP oligomerization and enzymatic activity, as well as preventing nuclear translocation of PKM2 and its role as a transcriptional coactivator leads to decreased glycolysis and impacts HCC cell proliferation and metastasis (Yu and Li, 2017; Guan et al., 2020). LINC01554 mediates glycolysis, and its downregulation in HCC suppresses tumorigenesis. LINC01554 also promotes the ubiquitination-mediated degradation of the glycolytic enzyme PKM2 and inhibits Akt/mTOR signaling to reduce glycolysis in HCC cells. Increased levels of miR-365a have also been found to reduce LINC01554 expression. Most importantly, the results of one of these studies showed that a nude mouse model of tumors formed by LINC01554-transfected cells were reduced by the Akt inhibitor MK2206. Based on these findings, LINC01554 has the potential as a prognostic marker in Akt inhibitor-treated patients with HCC (Zheng et al., 2019).

Fat metabolism is also important in HCC progression. By controlling the expression of Src homology 2 domain-containing protein tyrosine phosphatase 1 (SHP-1), a protein involved in glucose and lipid metabolism in the liver, hepatocyte nuclear factor 1α (HNF1α) inhibits HCC. To slow the development of HCC, sorafenib boosts SHP-1 activity and inhibits EMT produced by TGF-β (Shih et al., 2001; Dubois et al., 2006; Lopez-Ruiz et al., 2011; Tai et al., 2012; Xu et al., 2014; Fan et al., 2015). The lncRNA HNF1A-AS1 has been identified to have a role in HNF1’s anti-HCC actions. It has been shown that hepatic nuclear factor 1 (HNF1) binds to the HNF1A-AS1 promoter, which in turn stimulates the production of HNF1A-AS1. To suppress HCC, HNF1A-AS1 acts as a phospho-activator, connecting directly with SHP-1 to boost its phosphatase activity (Ding et al., 2018). As shown by Liu et al. lncRNA NEAT1 is involved in abnormal lipolysis. NEAT1 levels are increased in HCC where they suggest poor prognosis. NEAT1 competes with miR-124-3p for binding and then promotes increased expression of adipose triglyceride lipase (ATGL), which disrupts lipolysis in HCC cells, and it exerts pro-tumor effects by promoting diacylglycerol (DAG) and free fatty acid (FFA) production. ATGL can also promote the upregulation of the oncogene PPARα in HCC and promotes proliferation in HCC cells (Liu et al., 2018).

### Cell repair

Double-strand breaks (DSBs) represent the most serious form of DNA damage. Repair of DSBs occurs through homologous recombination (HR) and non-homologous end joining (NHEJ). In these two pathways, many oncogenes have been found to be involved in DSB repair signaling and may mediate malignancy (Khanna and Jackson, 2001). These authors identified a novel HR-promoting factor, the lncRNA PRLH1, which was upregulated in p53-mutated HCC samples and repressed by p53. PRLH1 can specifically bind to the DNA repair protein RNF169, forming an HR-repair functional complex, and promote the recruitment and retention of RNF169 at DSB sites, thereby promoting HCC HR repair and increasing proliferation in HCC cells (Deng et al., 2019). LncRNA NIHCOLE (lncRNA LINC02163) can target the NHEJ repair mechanism by binding to effector proteins promote the ligation efficiency of DSBs, thereby maintaining proliferation (Unfried et al., 2021).

## Relationships between lncRNAs and the tumor microenvironment in hepatocellular carcinoma

### Stem cells

Tumor cells that may self-replicate and differentiate in several ways are called cancer stem cells (CSCs) (Nassar and Blanpain, 2016). Evidence suggests that CSCs are important drivers of HCC growth and represent one of the reasons for cancer recurrence and drug resistance after chemotherapy/radiotherapy (Wang and Jacob, 2011). Markers of liver CSCs have been linked to poor patient prognosis. Lee et al. investigated the research status of related CSCs markers and related stem cell regulatory factors. These tumor markers identify distinct stem cell lineages that promote HCC development and characterize important mechanisms by which cancer stem cell-intrinsic regulators contribute to the tumor-initiating potential of HCC (Lee et al., 2022). There are many abnormally expressed lncRNAs in HCC, which not only play important roles in tumorigenesis but also maintain the characteristics of HCC stem cells (Huo et al., 2017).

A recently identified lncRNA, HAND2-AS1, is strongly expressed in hepatic CSCs. HAND2-AS1 has been found to promote self-renewal in liver CSCs and HCC development through the bone morphogenetic protein (BMP) signaling pathway. HAND2-AS1 binds the INO80 complex, recruiting it to the type IA bone morphogenetic protein receptor (BMPR1A) promoter, thereby inducing BMPR1A expression and activating BMP signaling (Wang et al., 2019c). Liu W. et al. (2020), using lipopolysaccharide induction of hepatic progenitor cells to fibroblasts, observed an increased risk of hepatic progenitor cell (HPC) carcinogenesis. Lipopolysaccharides/Toll-like receptor 4 (LPS/TLR4) signaling mediates HPC development into myofibroblasts that show high levels of IL-6 and TNF-α, potentially promoting upregulation of EGFR and downregulation of PTEN through lncRNA regulation, leading to abnormal Ras and p53 signaling and, ultimately, inducing the malignant transformation of HPCs, potentially leading to HCC development.

### EMT/metastasis

The EMT (Epithelial to Mesenchymal Transition) has been lined with various aspects of malignancy, including metastasis and drug resistance (Pastushenko and Blanpain, 2019). It is particularly associated with tumor metastasis (Bakir et al., 2020).

Increased lncRNA-MUF levels are associated with a dismal outlook in patients with HCC. This EMT is stimulated by LncRNA-MUF binding to annexin A2 (ANXA2), which in turn activates Wnt/β-catenin. Elevated expression of lncRNA-MUF has been linked to EMT in HCC-associated mesenchymal stem cells (HCC-MSCs) (Yan et al., 2017). LINC01133 is upregulated in HCC, interacting with annexin A2 (ANXA2) and activating ANXA2/STAT3/cyclinD1 signaling which, in turn, promotes HCC progression. LINC01133 also sponges miR-199A-5p to upregulate SNAI1, thereby promoting the EMT (Yin et al., 2021). HCC and lung metastases showed upregulation of the lncRNAs SNHG10 and its homolog SCARNA13. SNHG10 was discovered to boost c-Myb activity *via* stabilizing ribosomal protein L4 (RPL4) mRNA and promoting RPL4 expression, both of which were previously thought to occur independently of one another. Therefore, SNHG10, SCARNA13, and their downstream effector SRY-related high-mobility-group box 9 (SOX9) were all upregulated in HCC as a result of this. Cancer cells overexpressing SOX9 are known to exhibit increased cell proliferation and EMT (Lan et al., 2019b). Increased expression of lncRNA HCCL5 was reported by Peng et al., a functional lncRNA, in a TGF-β1-induced EMT model. When Zinc-finger E-box binding protein 1 (ZEB1) reached the HCCL5 promoter and super-enhancer, it immediately bonded to them and began driving transcription of that gene. HCCL5 promotes EMT progression in HCC cells by regulating EMT-related transcription factors (Peng et al., 2019). LncRNA HOXD-AS1 levels are significantly raised in metastatic HCC, where it appears to upregulate Rho GTPase-activating protein 11A (ARHGAP11A) through competitive binding to miR19a. HOXD-AS1 overexpression also downregulated regulator of G-protein signaling 3 (RGS3), leading to reduced doxorubicin (Dox)-induced apoptosis. Interestingly, RGS3 has been suggested to negatively regulate MEK/ERK signaling, and HOXD-AS1 is known to reduce RGS3 expression, which can block HCC cell apoptosis and promote proliferation through activation of MEK/ERK signaling (Lu et al., 2017). LncRNA LINC01278 levels are elevated in HCC where it negatively modulates the expression of miR-1258, thereby upregulating Smad2/3 and promoting HCC metastasis. Wnt/β-catenin signaling was shown to be involved in the regulation of LINC01278 by these authors as well. Elevated levels of β-catenin expression in HCC cells cause T cell factor 4 (TCF-4) to bind to the LINC01278 promoter, resulting in the overexpression of this gene. As with TGF-β/Smad signaling, miR-1258 has been shown to target Smad2/3 and suppress it. Based on these findings, LINC01278 may play a role in HCC metastasis by mediating the Wnt/β-catenin and TGF-β/Smad pathways (Huang W. J. et al., 2020). To facilitate -mediated m6A methylation of the GATA3 pre-mRNA 3′UTR, Lan et al. discovered a lncRNA, GATA3-AS, which interacts with KIAA1429 and the GATA3 pre-mRNA. HuR acted as a negative regulator of GATA3 expression by binding to GATA3 pre-mRNA, which decreased GATA3 expression and promoted HCC (Lan et al., 2019a). LncRNA 34a is also upregulated in HCC and has been associated with bone metastasis. While miR-34a targets Smad4 through the TGF- pathway to suppress HCC malignancy and bone metastasis, LncRNA 34a epigenetically represses miR-34a production by inducing methylation of the miR-34a promoter and histone deacetylation in HCC cells. This inhibitory effect of miR-34a was prevented by lncRNA 34a, which in turn mediates HCC bone metastasis (Zhang L. et al., 2019).

### Exosomes

Exosomes have been studied extensively and shown to have a role in invasion, migration, and EMT in a variety of malignancies (Bebelman et al., 2018; McAndrews and Kalluri, 2019; Dai et al., 2020).

The exosome secretion process begins when multivesicular bodies (MVB) fuses with the plasma membrane, releasing intraluminal membrane vesicle (ILV). The SNARE proteins are the mediators of MVB-membrane fusion. LncRNA HOTAIR is upregulated in HCC where it promotes exosome secretion. According to the work of Yang et al. (2019), HOTAIR promotes co-localization of vesicle-associated membrane protein 3 (VAMP3) and synaptosome associated protein 23 (SNAP23) to affect the development of the SNARE complex, leading to MVB fusion with the plasma membrane. Additionally, phosphorylation of SNAP23 by HOTAIR stimulates exosomal release.

## Application of lncRNAs in the clinical diagnosis and treatment of hepatocellular carcinoma

Recent years have seen several breakthroughs in the ability to detect and diagnose HCC (Ye et al., 2019; Ahn et al., 2021; Nault and Villanueva, 2021; Zhang et al., 2021). lncRNAs are suitable choices for biomarkers. Liquid biopsy is an emerging testing modality that is being developed. Compared with traditional tissue biopsy, liquid biopsy can repeatedly obtain tumor samples and dynamically monitor the overall tumor status, reducing the bias caused by tumor heterogeneity (Heitzer et al., 2019; Ye et al., 2019; Moldogazieva et al., 2021). Saliva testing is an important aspect of liquid biopsy and has the advantage of being non-invasive and easy to collect. Research has found that salivary lncRNA lnc-PCDH9-13:1 was found to show more powerful diagnostic sensitivity and specificity than serum AFP and could be used as a specific biomarker for early and AFP-negative HCC (Xie et al., 2018). Extracellular vesicles are now also thought to mediate the process of cancer development. An optimized human plasma exLR-sequencing (exLR-seq) method has recently been proposed for the detection of long RNAs, including lncRNAs, in extracellular vesicles (EVs). Blood exLR heterogeneity may serve as biomarkers for HCC diagnosis and the development of an exLR profile based on exLR liquid biopsy may differentiate cancer patients from healthy persons (Li et al., 2019). HCC is characterized by elevated levels of the lncRNA NONHSAT122051, which is favorably connected with clinicopathological characteristics (such as poorly differentiated tumor, PVTT, and AFP levels) (Yao et al., 2017). In addition, PXN-AS1-IR3, a lncRNA, was shown to have increased levels in both HCC tissues and the serum of extrahepatic metastases (Zhou H. et al., 2022). This suggests that in the future, we can use liquid biopsy to identify these abnormal lncRNAs and determine the malignancy and clinical features of HCC based on this.

Drug resistance is a significant challenge to cancer chemotherapy, and the acquisition of tumor drug resistance is associated with tumor heterogeneity interacting with the surrounding microenvironment (Bhattacharya et al., 2021). Regarding anticancer drug resistance, Nussinov et al. (2021) described the research status and molecular mechanisms in detail, showing that lncRNAs were also involved in HCC drug resistance.

Progression and a poor prognosis in HCC are directly connected to resistance to chemotherapeutic treatments. A lncRNA, PDIA3P1, which regulates the chemoresistance of tumor cells, shows upregulation in HCC and is also highly expressed after antitumor treatment with DNA-damaging agents. Since PDIA3P1 acts as a ceRNA by binding to miR-125a/b and miR-124 to upregulate TRAF6 expression and enhance NF-kB signaling to suppress cancer cell apoptosis, its overexpression confers chemoresistance to HCC cells. Additionally, exosomes were found to mediate the degradation of PDIA3P1. The increase in PDIA3P1 levels induced by the DNA-damaging agent Dox was reduced when hMTR4, an important cofactor of exosomes, was overexpressed. These results provide additional evidence that targeting PDIA3P1 is critical for increasing the sensitivity of HCC to chemotherapeutics, and they show that exosomes play a role in the lncRNA PDIA3P1-mediated response to DNA damage (Xie et al., 2020). LncRNA ZFAS1 was observed to be significantly upregulated in HCC and recurrent tumors. Treatment with sorafenib leads to ZFAS1-induced promotion of stemness and normal EMT functioning in HCC, and it is possible that sorafenib-resistant stem-like cells may be present and may, after prolonged exposure to the drug, multiply and lead to overall drug resistance. Therefore, the elimination of quiescent CSC-like cells or silencing ZFAS1 could prevent sorafenib resistance and prolong survival in HCC patients (Zhou K. et al., 2022).

Many lncRNAs have been shown to be associated with increased tumor burden and poor prognosis based on their differential expression between normal and malignant tissues, and these lncRNAs are preferentially expressed in healthy testes or brain, where they are predicted to be oncogenes. Perhaps, in targeting these up-regulated lncRNAs with oncogenic potential, it is possible to comprehensively consider tissues that preferentially express these targets, and develop more comprehensive targeted therapy regimens to reduce the occurrence or progression of cancer (Unfried et al., 2019). Based on an RNA-binding protein (RBP)-centered approach, these authors identified a novel lncRNA, LINC00326, which is minimally expressed under normal physiological conditions and is speculated to act as an inhibitor to prevent the transformation of normal hepatocytes to malignant cells (Søndergaard et al., 2022). Zuo et al. (2020) identified a lncRNA, LINC00958, with elevated levels in HCC. The authors exploited tumor promotion by the LNC00958/miR-3619-5p/HDGF axis in HCC to design a novel nanoplatform for systemic therapy of HCC in which LINC00958 siRNA was encapsulated. This nanosystem significantly reduces tumor burden and prolongs survival, indicating that lncRNAs have great potential in using nanotechnology to treat malignant tumors.

The use of nanoplatforms to treat diseases is widely studied, and nanocomplexes targeting lncRNAs hold a wide range of promise (van der Meel et al., 2019). siRNA tumor-targeting nanocomplexes carrying the lncRNA MALAT1 not only inhibit the malignant behavior of glioblastoma multiforme (GBM) cells, but when combined with temozolomide (TMZ), they also increase the chemosensitivity of TMZ and improve the prognosis of GBM patients (Kim et al., 2018). A study shows that targeted delivery of ASO to the brain using glucose-encapsulated polymeric nanocarriers can reduce target lncRNA expression in the brain and treat central nervous system disorders (Min et al., 2020). Precise delivery of lncRNA lncAFAP1-AS1 siRNA using a nanoparticle (NP) platform improves the effectiveness of radiation therapy for triple-negative breast cancer (TNBC), which provides a rationale for combining targeted lncRNA therapy with radiotherapy (Bi et al., 2020). A combination therapy that co-writes nanoparticles, small interfering RNA/antisense oligonucleotide (siRNA/ASO) and lncRNA into transplanted neural stem cells (NSC), which targets the lncRNA Pnky and prevents Pnky-mediated differentiation of NSC into astrocytes and loss of therapeutic effect. This shows that nanomedicines targeting lncRNAs can enhance the great potential of stem cell-based treatments for stroke (Lin et al., 2021). Zuo et al. (2020) identified a lncRNA, LINC00958, with elevated levels in HCC. The authors exploited tumor promotion by the LNC00958/miR-3619-5p/HDGF axis in HCC to design a novel nanoplatform for systemic therapy of HCC in which LINC00958 siRNA was encapsulated. This nanosystem significantly reduces tumor burden and prolongs survival. Summarize the above, lncRNAs have great potential in using nanotechnology to treat malignant tumors.

Targeting risk factors for HCC and treating patients with HBV infection is also an important treatment pathway. Positive results from phase IIb clinical trials of the drug bepirovirsen, which is being developed against HBV. Significant reductions in HBsAg and HBV DNA were seen in treated CHB (Chronic Hepatitis B) patients, which contributed to improved clinical outcomes. Bepirovirsen is an ASO that specifically recognizes HBV mRNA and recruits the liver’s own enzymes to eliminate HBV-derived RNA, HBV DNA and viral proteins, achieving a functional cure for CHB patients, which helps avoid HCC (Yuen et al., 2021).

As one of the important types of solid tumors, the abnormal construction of extracellular matrix (ECM) is closely related to its development in HCC, where collagen, a major component of ECM, mediates the carcinogenic effects of HCC (Feng et al., 2017). One study found that aspirin (ASA), an anti-inflammatory drug, targets P4HA2, a key enzyme in collagen formation, to block collagen deposition. Further, lncRNA LMCD1-AS1 was found to target miRNA let-7g, which induced upregulation of target gene P4HA2 expression at post-transcriptional level, and P4HA2 induced liver fibrosis. Since the development of HCC is often associated with fibrosis, targeting the fibrotic process in HCC can be a good therapeutic strategy (Wang T. et al., 2019). A 26-year clinical follow-up study also provides support that regular, long-term aspirin use reduces the risk of HCC (Simon et al., 2018).

## Conclusion

Advances in science and technology have led to reductions in both the incidence and mortality of HCC. Nevertheless, HCC remains one of the most deadly cancers in the world. Therefore, improving the survival rate of HCC patients is an urgent need. For early-stage HCC, surgical resection or local therapy is usually recommended, while for intermediate-advanced HCC, transarterial chemo- and radioembolization (TACE/TARE) or systemic therapy is recommended (Heimbach et al., 2018). Therefore, for early-stage patients, we need to effectively exclude risk factors and improve HCC detection, leading to early diagnosis and early treatment. For patients with advanced disease, we should try our best to prolong the patient’s life and improve their quality of life. Currently, the most commonly used imaging method for monitoring HCC is CT/MRI, and the most effective biological method is measurement of blood AFP levels. However, this is far from enough, and new methods for the diagnosis and treatment of HCC are needed (Kanwal and Singal, 2019).

In the past, lncRNAs were considered transcriptional “noise” as they do not encode proteins, and were thought to have no real biological function (Louro et al., 2009; Rosenbloom et al., 2012). However, with the discovery of the first lncRNA H19 (Brannan et al., 1990; Gabory et al., 2009), the subsequent identification of lncRNA XIST mediation of X-chromosome inactivation (Brown et al., 1991), and advances in high-throughput sequencing, a large number of lncRNAs have been discovered (Iyer et al., 2015). These lncRNAs not only act as regulators to participate in the whole process of gene expression regulation (Zhang Y. et al., 2018; Cao et al., 2019; Qian et al., 2019; Gao et al., 2021), but also have the potential to encode functional peptides (Choi et al., 2019). In both the malignant tumor itself and its tumor microenvironment, and even in cell-to-cell communication, lncRNAs act as bridges to transmit information, and can guide cancer development and progression, mediating cancer cell proliferation, invasion, metastasis, and chemoresistance (Zhang et al., 2020a; Wang et al., 2020; Liu et al., 2021; Xu et al., 2022). In short, lncRNAs have great research prospects. Whether functioning as oncogenes or tumor suppressors, tumor biomarkers can be studied to identify associated targets in signal transduction pathways regulated by these lncRNAs. In conclusion, the lncRNA research on HCC is promising.

Regarding the early detection of HCC, liquid biopsy method can be used to detect circulating tumor cells (CTCs) and cell-free DNA (cfDNA). This helps with monitoring HCC (Chen V. L. et al., 2020) and has implications for the study of lncRNAs in HCC. Systemic therapy for patients with advanced HCC, including chemotherapy, targeted therapy, and immunotherapy, has also developed rapidly in the past few years (Llovet et al., 2018). Of course, this is inseparable from the study of cancer signaling pathways. However, due to the high morbidity and mortality of HCC and its drug resistance, we urgently need a new drug that overturns the traditional treatment to improve or even cure HCC patients. lncRNA drugs are the trend of future research. To date, due to the large number of LncRNAs, the variety of regulatory modalities, and the difficulties in targeting delivery and related immune responses, no clinical translation has been completed, and no HCC drugs or clinical trials have yet been conducted using lncRNAs as targets. It is promising that with the continuous development and intelligent transformation of nanotechnology, siRNA drugs, antisense nucleotide drugs, miRNA drugs, etc. can be developed by targeting lncRNAs in the future and delivering drugs to target lesions stably and precisely through nanoplatforms, which not only has great potential but also needs to face great challenges (de Lázaro and Mooney, 2021; Kumar et al., 2021; Mast et al., 2021).
